# Incidence of Alpha-Globin Gene Defect in the Lebanese Population: A Pilot Study

**DOI:** 10.1155/2015/517679

**Published:** 2015-03-05

**Authors:** Chantal Farra, Rose Daher, Rebecca Badra, Rym el Rafei, Rachelle Bejjany, Lama Charafeddine, Khalid Yunis

**Affiliations:** ^1^Department of Pathology & Laboratory Medicine, American University of Beirut Medical Center, Beirut, Lebanon; ^2^Department of Pediatrics & Adolescent Medicine, American University of Beirut Medical Center, Beirut, Lebanon

## Abstract

*Background*. It is well established that the Mediterranean and Arab populations are at high risk for thalassemias in general and for alpha-thalassemia in particular. Yet, reports on alpha-thalassemia in Lebanon are still lacking. In this study, we aim at assessing the incidence of alpha-thalassemia in the Lebanese population. *Methods*. 230 newborns' dried blood cards remaining from routine neonatal screening at the American University of Beirut Medical Center were collected for DNA extraction. Samples were screened for the 21 most common *α*-globin deletions and point mutations reported worldwide, through multiplex Polymerase Chain Reaction (PCR) and Reverse-Hybridization technique. *Results*. Upon analyses, the carrier rate of *α*-thalassemia was found to be 8%. Two mutations detected the −*α* ^3,7^ single gene deletion found in 75% of cases and the nongene deletion *α*2 IVS1 [−5nt] in the remaining samples. *Conclusion*. This study is the first dedicated to investigate *α*-thalassemia trait incidence in Lebanon. Data obtained demonstrates a high carrier rate in a relatively, highly consanguineous population; it also highlighted the presence of two common mutations. These results may be of an important impact on premarital and newborn screening policies in our country.

## 1. Introduction

Alpha-thalassemia is one of the most common hemoglobin gene defects. It is considered as a severe, life-shortening autosomal recessive disease [1] which is caused by downregulation of *α*-globin synthesis with underproduction of fetal (HbF, *α*_2_ *γ*_2_) and adult (HbA, *α*_2_ *β*_2_) hemoglobin. The clinical phenotype in alpha-thalassemia varies according to the number of *α*-genes affected [2].

Four clinical conditions of variable severity are recognized: the silent carrier state (−1 gene), the alpha-thalassemia trait (−2 genes), the intermediate form of hemoglobin H disease (−3 genes), and the hemoglobin Bart hydrops fetalis syndrome which is lethal in utero or soon after birth (−4 genes) [2].

**Figure 1:**
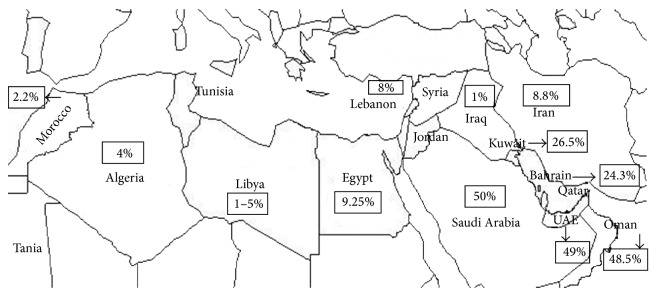
*α*-thalassemia carrier frequency in some Eastern Mediterranean countries.

The Mediterranean and Arab countries are considered high risk areas for thalassemia in general and for *α*-thalassemia in particular where the frequency exceeds by far that of beta-thalassemia in some of these populations. Alpha-thalassemia carrier frequency can vary among countries to reach the highest in UAE, Oman, and Saudi Arabia with a 50% carrier rate [3] (Figure 1). While no effective treatment for thalassemia has been reported to date, studies have shown that carrier screening and genetic counseling are the most effective solutions for reducing its incidence. Indeed, prenatal as well as premarital screening programs in countries with high incidence have already been established. In Lebanon; accurate epidemiological data on alpha-thalassemia is still lacking.

In this study, we screened for *α*-globin gene defects in a sample of Lebanese newborns at the American University of Beirut Medical Center over a period of one year.

## 2. Methods

Two hundred and thirty Lebanese newborns (age 1–3 days) at the AUBMC from the period of August 2012 to July 2013 were included in this research. The study was approved by the Institutional Review Board (IRB) of the AUB. AUBMC is a quaternary care center that receives referrals from all around Lebanon. Parental approval was obtained after explaining to them the risks and benefits of participation in this research study that serves as a basis to design systematic study to assess the incidence of disease causing *α*-globin gene alleles in our population. Remaining blood samples from the newborn's blood cards that are received routinely in the Chemistry Department of laboratory medicine for routine G6PD screening in neonates were collected. DNA was extracted from the blood collection cards, using the QIAamp DNA Micro Kit (Qiagen) and as described by the manufacturer. DNA analysis for the identification of the following 21 *α*-globin mutations, 3.7 and 4.2 single gene deletions, MED, SEA, THAI, FIL, and 20.5 double gene deletions, anti-3.7 triplication, *α*1 cd 14, *α*1 cd 59, *α*2 init cd, *α*2 cd 19, *α*2 IVS1 [−5nt], *α*2 cd 59, *α*2 cd 125, *α*2 cd 142 (Hb Constant Spring), *α*2 cd 142 (Hb Icaria), *α*2 cd 142 (Hb Pakse), *α*2 cd 142 (Hb Koya Dora), *α*2 poly A-1, and *α*2 poly A-2, was performed by means of Polymerase Chain Reaction (PCR) followed by Reverse-Hybridization techniques. Three multiplex PCR reactions were performed for each sample following the manufacturer's instructions (Viennalab, GmbH Vienna, Austria) and as previously described [4].

Data analysis was performed using Statistical Package for Social Sciences (SPSS) version 19. Descriptive statistics was done using frequencies and percentages.

## 3. Results and Discussion

Alpha-thalassaemia occurs at high frequencies in parts of the world extending from sub-Saharan Africa through the Mediterranean region and Middle East, to South East Asia with a carrier frequency reaching as high as 80–90% in some of these populations such as Nepal and Andhra Pradesh province of India [5].

Although numerous studies have been carried out to assess the carrier rate of *β*-thalassemia in Lebanon (2-3%) [6], data on *α*-thalassemia is still incomplete.

In this pilot study a total of 230 neonates were recruited in total: 30 of them were rejected due to inadequate blood amount. Of the 200 DNA samples analyzed, 8% were found to be carriers of one *α*-gene defect. This is much higher compared to carrier rate of beta-thalassemia in our population (2%) [6]. Our finding is comparable to many Middle Eastern and Mediterranean countries such as Israel (5–9%) [7], Turkey (7.5%) [8], and Greece (7%) [9] but lower than that reported from the countries in the Arabian Peninsula (Figure 1).

Only two alpha-thalassemia gene defects have been observed in our newborn carriers. These are −*α* ^3,7^ (75%) and *α*2 IVS1 [−5nt] (25%). −*α* ^3,7^, the most common, is a well described founder mutation in the Mediterranean and Middle Eastern countries [3, 7, 10–12]. *α*2 IVS1 [-5nt] is also highly prevalent in the Mediterranean countries [10] but is rarely detected in the Arabian Peninsula, whereas the *α*-thalassemia determinants - -^20.5^ and −^MED^, relatively common among both Middle Eastern and Mediterranean countries, were not detected in our sample (Table 1). In addition, *α*polyA1*α*, an equally common mutation in the Arabian Peninsula [10], was not detected in our population.

Studies have shown that the incidence of alpha-thalassemia vary among different ethnic groups and communities [13]. Lebanon is composed of a population with heterogeneous background and where intercommunity mating is still a rare occurrence. In our study, out of the 200 newborns, 136 were Muslim (68%), 57 were Christian (28.5%), and 7 were Druze (3.5%). We found that the carrier rate of alpha-thalassemia in the Muslim community (9.6%) is almost twice that of the Christian community (5.3%). No deleterious alpha-thal gene was found in the Druze families analyzed. The small number of Druze families that were recruited (*n* = 7) reflects our population's constitution with the Druze community representing less than 5% of the total Lebanese population [14].

Allelic distribution did not differ between communities with −3,7del single gene deletion being the most common in both Muslims and Christians.

## 4. Conclusion

This study is the first dedicated to investigate *α*-thalassemia trait incidence in Lebanon. The high incidence of alpha-globin gene defect found should steer attention to the importance of hemoglobinopathies in our population and may justify contemplating social preventive measures such as establishing premarital, screening for *α*-thalassemia especially in consanguineous mating.

Also based on our findings, inclusion of molecular diagnosis for *α* ^3.7^ and *α*2 IVS1 [−5nt] determinants in the general newborn screening should be envisaged. This will serve for an early, adequate management of hemoglobinopathies in our population.

## Figures and Tables

**Table 1:** The type and frequency of *α*-gene mutations detected by ethnicity.

	Muslim	Christian	Druze
Total *n* (%)	136 (68%)	57 (25.8%)	7 (3.5%)
Mutations (%)	13 (9.6%)	3 (5.3%)	0 (0%)
−α^3,7^	*10 (77%) *	*2 (67%) *	*— *
*α*2 IVS1 [−5nt]	*3 (23%) *	*1 (33%) *	*— *
